# Depressive symptoms—Not a predictor for five-year mortality in patients with subjective cognitive decline, non-amnestic and amnestic mild cognitive impairment

**DOI:** 10.1007/s40211-024-00495-2

**Published:** 2024-05-22

**Authors:** Alexander Gerschmann, Johann Lehrner

**Affiliations:** Department of Neurology, Medical University of Vienna, Währinger Gürtel 18- 20, 1090 Vienna, Austria

**Keywords:** 5‑year mortality, aMCI, naMCI, SCD, Depression, Neurocognitive function, 5-Jahres-Sterblichkeit, aMCI, naMCI, Depression, Neurokognitive Funktionen

## Abstract

The main aim of the present study is to evaluate the influence of depressive symptoms on mortality in patients with SCD (subjective cognitive decline), naMCI (non-amnestic mild cognitive impairment), and aMCI (amnestic mild cognitive impairment). Additional factors (age, sex, years of school attendance, and neuropsychological performance) were considered to determine the impact on survival probability. A monocentric retrospective data analysis based on adjusted patient protocols (*n* = 1221) from the observation period 1998–2021, using the Cox Proportional Hazards model, assessed whether depressivity had an explanatory value for survival, considering SCD as the reference level in relation to naMCI and aMCI. Covariates were included blockwise. Cox regression revealed that depressiveness (Beck Depression Inventory, Geriatric Depression Scale) did not make a significant contribution as a risk factor for mortality in all five model blocks, BDI-II with HR 0.997 [0.978; 1.02] and GDS-15 with HR 1.03 [0.98; 1.08]. Increasing age with HR 1.09 [1.07; 1.11] and male sex with HR (inverted) 1.53 [1.17; 2.00] appeared as risk factors for increased mortality across all five model blocks. aMCI (vs. SCD) with HR 1.91 [1.33; 2.76] showed a significant explanatory value only up to the fourth model block. By adding the six dimensions of the Neuropsychological Test Battery Vienna in the fifth model block, the domains attention and perceptual speed with HR 1.34 [1.18; 1.53], and executive functions with HR 1.24 [1.11; 1.39], showed substantial explanatory values for survival. Accordingly, no tendency can be attributed to depressiveness as a risk factor on the probability of survival, whereas the influence of certain cognitive dimensions, especially attention and perceptual speed, and executive functions, can be seen as protective for survival.

## Abstract (de)

Das Hauptziel der vorliegenden Studie besteht darin, den Einfluss depressiver Symptome auf die Mortalität bei Patienten mit SCD (Subjective Cognitive Decline), naMCI (nonamnestic Mild Cognitive Impairment) und aMCI (amnestic Cognitive Decline) zu untersuchen. Zusätzliche Faktoren (Alter, Geschlecht, Bildungsjahre und neuropsychologische Leistung) wurden berücksichtigt, um den Einfluss auf die Überlebenswahrscheinlichkeit zu bestimmen. Eine monozentrische retrospektive Datenanalyse basierend auf 1221 Patientenprotokollen aus dem Beobachtungszeitraum 1998–2021 unter Verwendung des Cox Proportional Hazards-Modells untersuchte, ob Depressivität einen erklärenden Wert für das Überleben hatte, wobei die SCD-Gruppe als Referenz in Bezug auf naMCI und aMCI berücksichtigt wurde. Die Cox-Regression ergab, dass Depressivität (Beck Depression Inventory, Geriatric Depression Scale) in allen fünf Modellblöcken keinen signifikanten Beitrag als Risikofaktor für Mortalität leistete (BDI-II mit HR 0,997 [0,978; 1,02]) und (GDS-15 mitHR 1,03 [0,98; 1,08]). Chronologisches Alter mit HR 1,09 [1,07; 1.11] und männliches Geschlecht mit HR (invertiert) 1,53 [1,17; 2,00] waren Risikofaktoren für eine erhöhte Mortalität in allen fünf Modellblöcken. aMCI (vs. SCD) mit HR 1,91 [1,33; 2,76] zeigte nur bis zum vierten Modellblock einen signifikanten Erklärungswert. Die Domänen Aufmerksamkeit mit HR 1,34 [1,18; 1,53] und Exekutivfunktionen mit HR 1,24 [1,11; 1,39] hatten einen signifikanten Einfluss auf das Überleben. Zusammenfassend zeigte sich, dass Depressivität kein Risikofaktor für die Überlebenswahrscheinlichkeit darstellt. Die kognitiven Dimensionen, Aufmerksamkeit und exekutive Funktionen, hatten einen protektiven Einfluss auf das Überleben.

## Introduction

Due to an increasingly ageing society, age-related concomitants are becoming more relevant [1]. Dementia is the most advanced stage of cognitive decline. In clinical practice, it is expressed as cognitive deficits that affect daily functioning and lead to loss of patient autonomy [2]. Mild cognitive impairment (MCI) is also classified as objective cognitive impairment. In contrast to patients with dementia, the daily functioning of MCI patients remains broadly intact, and independence is retained [3]. A distinction is made between the non-amnestic form of MCI (naMCI) and the amnestic form of MCI (aMCI), as each form is associated with different etiologies and clinical presentations in terms of outcome. aMCI is traditionally the prodromal stage of dementia [4]. An increasing number of patients recognize a subjective reduction in cognitive function, but their neurocognitive abilities do not show any proof of objective cognitive decline by cognitive testing or in daily practice. These individuals are classified in most cases as healthy. Due to this mismatch, subjective cognitive decline (SCD) can be understood as a transitional phase in the progression from normal cognitive function to upcoming cognitive decline [5, 6], with a future tendency to MCI in 6.6% and to dementia in 2.3% [7].

The current state of studies clearly shows an association between higher mortality in older people and moderate cognitive impairment or Alzheimer’s disease [8–10]. In addition, other co-variables such as age, gender, functional limitations, and psychiatric syndromes such as depression have an impact on the mortality in people with cognitive decline. According to the literature, the severity of dementia-related developments has an inconsistent effect on survival, but the predictive role of given individual characteristics depends on the degree of cognitive decline [11]. In comparison, it could be demonstrated that an advanced state of cognitive decline is related to an increased mortality rate and a reduced time of survival [12].

Cognitive decline and depressiveness are common psychiatric syndromes in old age [13], especially SCD which often occurs in association with depression [5, 6]. The correlation between depression and dementia derives from related biological mechanisms such as vascular disease, glucocorticoid steroid alterations and hippocampal atrophy, increased beta-amyloid plaque deposition, inflammatory changes and nerve growth factor deficits. [14]. Earlier onset of depression or depressive symptoms is related to a 2- to 4‑fold increased risk of dementia [15]. The association with depression in later life is less specific. For example it is not clear whether depression is a prodrome, consequence or risk factor for dementia. [14]. Even though depression and cognitive decline are treated as different clinical entities, they share some joint characteristics: impairment in attention and working memory, alterations in sleep patterns, and a decrease in social and occupational function [13].

The research on the relationship between depression and cognitive decline is addressed, but few studies show the impact of depressive symptoms on mortality in patients with SCD, naMCI, and aMCI. The aim of the present study is to evaluate the clinical relevance of depressive symptoms in terms of survival in a group of patients with cognitive decline.

## Material and methods

The study is a university-based, single-center, retrospective data analysis. Data were derived from patients of the Neurological Department of the Medical University of Vienna from the period August 1998 to October 2021. Patient-related data of the deceased patients were obtained from the Research, Documentation, and Analysis (RDA) system of the Medical University of Vienna. Data of deceased patients up to March, 14th 2022 were accessed according to the records of the RDA and the Allgemeines Krankenhaus Information Management system (AKIM).

### Participants

Participants underwent a complete medical and neurological examination at the Department of Neurology. Individuals were included in the study if they were examined both for depression and dementia. Patients were divided into three subgroups of cognitive impairment, according to the precursors of Alzheimer’s dementia: SCD, naMCI, and aMCI. Patients without cognitive decline or a severe form such as Alzheimer’s dementia, evidence of stroke, traumatic head injury, and psychiatric syndromes (e.g. schizophrenia, severe anxiety disorders such as Bipolar Disorder, severe Personality Disorders such as Borderline Disorder, severe compulsive disorders) causing pseudo-dementia were excluded from the study. Exclusion criteria were assessed via clinical exploratory interview.

**Fig. 1:**
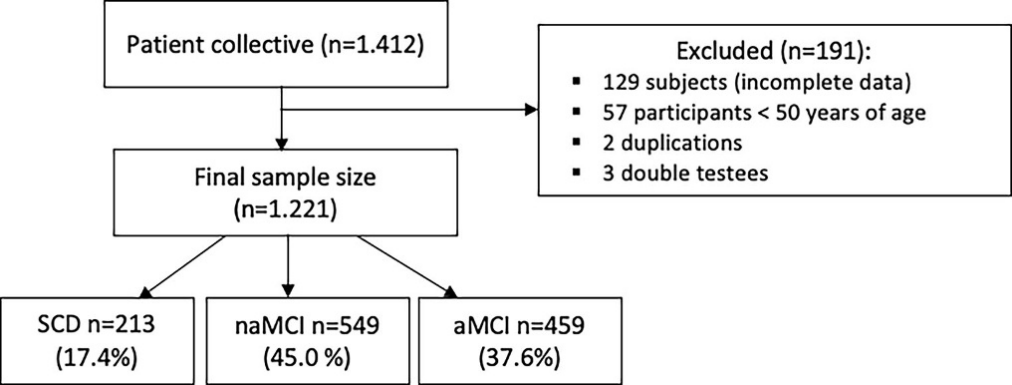
Flowchart, showing the selection of the patient collective based on the exclusion of cases from the study and the distribution of the remaining patients. *SCD* subjective cognitive decline, *naMCI* non-amnestic mild cognitive impairment, *aMCI* amnestic mild cognitive impairment

The diagnosis of SCD was made according to the classification of Jessen et al. based on self-reported concerns of cognitive problems. Individuals affected with SCD were not diagnosed until 1999 [16]. Similarly, Petersen’s criteria were applied to diagnose MCI patients [3]. Sociodemographic characteristics about age, gender, and average school attendance in years were considered. Neuropsychological abilities indicated by the Mini-Mental-State-Examination (MMSE), vocabulary test (WST-IQ) and the Neuropsychological Test Battery Vienna (NTBV) were used. The neuropsychological testing as well as the assessment of depressiveness by Beck Depression Inventory (BDI-II) and Geriatric Depression Scale (GDS-15) were conducted on the same day. Figure 1 illustrates the exclusion protocol for participants who did not meet the required criteria for the present study. With a total of 1221 cases, the sample was divided into 213 SCD, 549 naMCI, and 459 aMCI patients. The age range was between 50–90 years, as most of the memory clinic’s patients with cognitive decline belong to this age group. The study was approved by the ethics committee of the Medical University of Vienna (2193/2021) and followed the tenants of the Declaration of Helsinki.

### Instruments

#### Depressive symptoms

The Beck Depression Inventory (BDI-II) assesses the severity of depressive symptoms in a clinical setting. The BDI-II consists of 21 items, operationalizing sadness, pessimism, feelings of failure, loss of joy, feelings of guilt, feelings of punishment, self-rejection, self-reproach, suicidal thoughts, crying, restlessness, loss of interest, inability to make decisions, worthlessness, loss of energy, change of sleeping habits, irritability, change in appetite, difficulty concentrating, fatigue or exhaustion, and loss of sexual interest, based on a four-staged rating [17, 18]. The scoring range is as follows [19] < 13: no depression or clinically unremarkable or remitted; 13–19: mild depressive syndrome; 20–28: moderate depressive syndrome; ≥ 29: major depressive syndrome.

The Geriatric Depression Scale (GDS-15) is a widely used international assessment tool that provides reproducible information about the mental state of ageing patients. The 15 questions are answered with yes/no. The following scores are obtained after counting positively answered questions [20] 0–5 points: inconspicuous; 5–10 points: mild to moderate depression; 10–15 points: severe depression.

#### Neurocognitive status

Neurocognitive tests were used to determine the progression of the patient’s neurodegenerative processes. The Mini-Mental State Examination (MMSE), a brief assessment of cognitive performance, is often used as part of the screening and diagnosis of potential dementia [21]. It contains few items on episodic and semantic memory or spatial-visual reasoning and does not test executive function. Performance is impaired by age, ethnicity, and low educational level [22]. The Wortschatztest (WST/WST-IQ) provides a rapid assessment of verbal intelligence level and language comprehension. The average IQ value is between 85 and 115 IQ points (μ = 100, σ = 15) [23]. The Neuropsychological Test Battery Vienna (NTBV) is a standardized neuropsychological test series for the assessment of objective cognitive impairment and dementia. The inventory includes categories such as psychomotor speed, attention, language, memory, and executive functions. These areas of cognitive function are characteristically affected in Alzheimer’s dementia [24]. In the present study, 24 subtests of the NTBV were addressed in six domains. The NTBV can be obtained from www.psimistri.com [25].

### Statistical analysis

For the realization of the descriptive and inferential statistical analyses, IBM SPSS® 28.0 for macOS was used. The hypotheses testing was implemented under the assumption of an alpha level of 5% (α = 0.05), according to the Type-I-Error. The results in *p* ≤ 0.05 were considered significant in the analyses. All the statistical procedures were performed two-tailed. In the case of multiple testing, Bonferroni adjustment was considered to avoid the accumulation of type I errors.

For the characterization of metric parameters, mean (*M*) and standard deviation (*SD*), a range of minimum (min) and maximum (max) were used as key values. In case of skewed data distribution, the median (*Md*) and interquartile range (IQR, PR 25–75%) were assessed and reported. 95%-confidence intervals (CI) were generated specifying a range estimate [26]. For the description of nominal scaled parameters, the frequencies (n) and the proportional values (%) were calculated and displayed. In the inferential statistics section, variance analytic procedures (ANOVA) were calculated to test for differences in metric, at least interval-scaled, parameters between more than two groups [27]. In addition, the homogeneity of variances had to be considered, which was examined by Levene’s test. In presence of heterogeneous variances, Welch’s ANOVA was applied [28]. Non-parametric analyses, Mann-Whitney’s U‑testing, and the Kruskal-Wallis-Test were used for at least ordinally scaled data comparing independent groups [27]. The relationship between two nominally scaled variables was examined based on cross-tabulations using chi-square testing. The relationship between two metric, at least interval-scaled variables was calculated using Pearson’s coefficient *r*. For skewed variables, Spearman’s parameter-free rank correlation *r*_s_ was also applied [26].

Furthermore, the Kaplan-Meier function and the post-hoc log-rank procedure were applied to analyze the differences in survival among the different diagnosis groups [29]. The Cox Proportional Hazards model was used to evaluate the weight of depressive symptoms in predicting the mortality of patients with cognitive decline considering the time component; follow-up time until death and excluded cases. The hazard ratio (HR) was reported as a measure of the relative risk of a predictor. The influence of the severity of cognitive decline as well as the impact of covariates on the mortality of the patients was determined [30, 31]. These covariates were included hierarchically block-wise in five steps, to successively explain the additional value considering the added predictors using the so-called enter method per block [30]. In the 1st block, the depression scorings (BDI-II, GDS); in the 2nd block the sociodemographic covariates age (years), gender (0) *male*, (1) *female*; in the 3rd block education in years and WST-IQ were used; in the 4th block the diagnostic subgroups considering SCD as reference level; and finally in the 5th block the *z*-standardized factor scores of the six NTBV dimensions. Achieving a dimensional reduction and overview, the NTBV subtests were subject to a principal component analysis (PCA) with subsequent orthogonal rotation (varimax approach according to Kaiser) [32]. To fully assess the information content of the items, so-called factor scores were generated. The main advantages of this process are *z*-standardized scores (μ = 0, σ = 1) with perfect independence (*r* = 0) and complete uncorrelatedness of the factor scores [33–35].

## Results

Demographics, clinical and neurocognitive parameters data are summarized as baseline characteristics in Table 1, showing percentages and medians. The percentage of female participants (55.2%; 95%-CI [52.4%; 58.0%]) was proportional to the rate of women in the total population aged over 50 (53.1%) [36].

**Table 1:** Key values (Md; IQR), frequencies and percentages of the study relevant parameter considering diagnostic subgroups

Parameter	Diagnostic subgroup			Total *N* *=* 1221
	SCD *n* = 213	naMCI *n* = 549	aMCI *n* = 459	
Age (years) at testing	67.8 (60.4; 74.6)	68.4 (60.1; 74.6)	69.4 (61.6; 75.8)	68.7 (60.7; 75.0)
Age (years) at death	*n* = 38 (17.8%) 85.8 (77.5; 88.7)	*n* = 120 (21.9%) 83.5 (76.0;89.3)	*n* = 147 (32.0%) 80.7 (75.5;87.0)	*n* = 305 (25.0%) 82.5 (76.1; 88.5)
Gender male	105 (49.3%)	214 (39.0%)	228 (49.7%)	547 (44.8%)
Female	108 (50.7%)	335 (61.0%)	231 (50.3%)	674 (55.2%)
School att.(years)	12.0 (9.0; 16.0)	11.0 (8.0; 15.0)	11.0 (8.0; 16.0)	12.0 (8.0; 15.0)
BDI-II (0–63)	8.0 (5.0; 14.0)	10.0 (5.0; 16.0)	10.0 (5.0; 15.0)	9.0 (5.0; 15.0)
GDS-15 (0–15)	3.0 (1.0; 5.0)	3.0 (1.0; 6.5)	3.0 (1.0; 6.0)	3.0 (1.0; 6.0)
MMSE (0–30)	29 (28; 29.5)	28 (27; 29)	28 (27; 29)	28 (27; 29)
WST-IQ (μ = 100,σ = 15)	114 (107; 123.5)	110 (99; 118)	107 (97; 118)	110 (99; 118)
NTBV dimension	*n* = 213	*n* = 549	*n* = 459	*n* = 1221
F1 Attention & percept. speed^1^	−0.391 (−0.725; −0.034)	−0.036 (−0.621; 0.641)	−0.103 (−0.672; 0.706)	–
F2 Verbal memory	0.512 (0.008; 1.002)	0.492 (−0.017; 1.00)	−0.70 (−1.352; −0.196)	–
F3 Exec. Functions^1^	−0.232 (−0.123; 0.795)	−0.048 (−0.658; 0.610)	−0.009 (−0.668; 0.559)	–
F4 Planning capacity	0.232 (−0.123; 0.795)	0.056 (−0.653; 0.610)	−0.026 (−0.663; 0.660)	–
F5 Verbal fluency	0.382 (−0.023; 0.943)	−0.091 (−0.733; 0.574)	−0.109 (−0.766; 0.615)	–
F6 Divergent reasoning^1^	−0.337 (−0.662; −0.025)	−0.091 (−0.562; 0.502)	−0.198 (−0.680; 0.371)	–

^1^ reversed, negative signs of the reversed dimensions (F1, F3, F6) indicate higher performance

*aMCI* amnestic mild cognitive impairment; *naMCI* non-amnestic mild cognitive impairment; *SCD* subjective cognitive decline; *BDI-II* Beck-Depressions-Inventar II; *GDS* Geriatric Depression Scale; *MMSE* Mini Mental State Examination; *WST-IQ* Wortschatztest-IQ; *NTBV* Neuropsychological Test Battery Vienna

Differences between diagnostic subgroups considering p‑values are depicted in the following. The corresponding test values of the cross-tabulation revealed a significant distribution of patients’ sex regarding the diagnostic subgroups, *p* = 0.001, indicating a higher proportion of female patients (61.0%) within the naMCI group. The mean age of diagnostic subgroups did not reveal significant differences, *p* = 0.211. Participants’ years of school attendance between the diagnostic subgroups did not show a significant difference either, *p* = 0.437. Regarding the three diagnostic subgroups, a non-significant difference in life expectancy in years was considered, *p* = 0.090.

### Depressive symptoms

Characteristics of the depression parameters including BDI-II and the GDS-15 in each of the diagnostic subgroups did not reveal a significant difference in BDI-II data (*p* = 0.056) and in the GDS-15 (*p* = 0.062).

### Neurocognitive functions

MMSE performance showed significant differences between diagnostic subgroups, *p* < 0.001. Pairwise comparisons revealed significant differences between SCD vs. aMCI, SCD vs. naMCI, and aMCI vs. naMCI, each *p* *<* 0.001. Furthermore, WST-IQ also revealed significant differences in comparing the diagnostic subgroups, *p* ≤ 0.006.

In the next step, NTBV-24 subtest results were analyzed using a PCA, applying the varimax rotation method according to Kaiser’s normalization. The information criterion, KMO = 0.838, indicated a sufficient level of information to conduct a PCA, achieving a dimensional reduction, explaining 71.0% of variance. Naming and labeling of the independent dimensions were performed regarding the respective high-loading subtests, which are designated as marker variables. The subtests have been organized into six dimensions suggesting the following meta key terms, corresponding to Table 2: (1) Attention and perceptual speed, (2) Verbal memory, (3) Executive functions, (4) Planning capacity, (5) Verbal fluency and (6) Divergent reasoning. The rotation converged in 14 iterations and a six-dimensional solution may be suggested, showing good discriminatory power in distinguishing SCD from MCI, whereas the naMCI and aMCI subgroups only differ in verbal memory, with better abilities for the naMCI diagnostic subgroup. To assess the difference in performance on the six dimensions regarding the three diagnostic subgroups, one-way Welch-ANOVAs were performed to account for heterogeneous variances. Further the z‑standardized factor scores obtained were applied. It should be noted that the negative signs of the reversed dimensions (F1, F3, F6) indicate higher performance, as shown in Table 1.

**Table 2:** Rotated component matrix; factor loadings, communality of items and eigenvalue of identified dimensions (*n* = 1221)

NTBV subtests	Dimension (component, factor F)						Communality
	F1	F2	F3	F4	F5	F6	h_i_^2^
TMT‑B	*0.791*	−0.197	0.260	−0.210	−0.006	0.281	0.86
Interference (TMT-B-TMT-A)	*0.745*	−0.179	0.233	−0.080	0.022	0.360	0.78
AKT time	*0.686*	−0.128	0.184	−0.283	−0.081	−0.082	0.62
AKT total/time	*−0.667*	0.157	−0.183	0.340	0.169	0.101	0.66
Interference (c.I.) (time)	*0.658*	−0.077	0.425	−0.078	−0.289	−0.039	0.71
Symbols (c.I.)	*0.657*	−0.041	0.030	−0.207	−0.216	−0.138	0.54
HAWIE‑R	*−0.624*	0.298	−0.243	0.290	0.234	0.058	0.68
Interference (c.I.) (total/time)	*−0.605*	0.092	−0.414	0.093	0.341	0.073	0.68
Stroop color word test (time)	*0.563*	−0.114	0.247	−0.056	−0.441	−0.110	0.60
TMT‑A	*0.546*	−0.149	0.210	−0.458	−0.077	−0.062	0.58
VSRT Delayed Recall	−0.109	*0.870*	−0.125	0.133	0.124	−0.063	0.82
VSRT Total Recall	−0.202	*0.861*	−0.173	0.101	0.201	−0.026	0.86
VSRT Immediate Recall	−0.155	*0.772*	−0.090	0.035	0.187	−0.010	0.67
VSRT Recognition	−0.066	*0.715*	−0.047	0.068	−0.058	0.027	0.53
Stroop color word test interference	0.223	−0.149	*0.906*	−0.191	−0.030	0.063	0.93
Stroop color word test (words)	0.394	−0.165	*0.834*	−0.178	−0.193	0.012	0.95
Stroop color word test (total/time)	−0.334	0.179	*−0.801*	0.155	0.233	0.021	0.86
NAI Planning maze test (time)	0.359	−0.104	0.163	*−0.790*	−0.023	0.044	0.79
NAI Planning maze test (total/time)	−0.346	0.131	−0.122	*0.782*	0.076	−0.025	0.77
5‑point test	−0.418	0.189	−0.226	*0.454*	0.227	0.224	0.57
PWT	−0.317	0.175	−0.154	−0.006	*0.670*	−0.050	0.61
SWT	−0.330	0.351	−0.135	0.244	*0.590*	0.005	0.66
BNT	0.075	0.030	−0.162	0.471	*0.487*	−0.307	0.59
5‑point test Perseverations	−0.032	−0.026	−0.003	−0.011	−0.054	*0.853*	0.73
Eigenvalue (λ)	5.37	3.16	3.08	2.45	1.82	1.16	17.04
Explained variance component	22.4%	13.2%	12.8%	10.2%	7.6%	4.8%	71.0%

*AKT* Alters-Konzentrationstest; *BNT* Boston Naming Test; *NTBV* Neuropsychological Test Battery Vienna; *PWT* Phonematic Verbal Fluency Test; *VSRT* Verbal Selective Reminding Test; *SWT* Semantic Verbal Fluency Test; *TMT* Trail Making Test; *HAWIE‑R* Number Symbol Test

### Model for influencing factors on five-year mortality

**Fig. 2:**
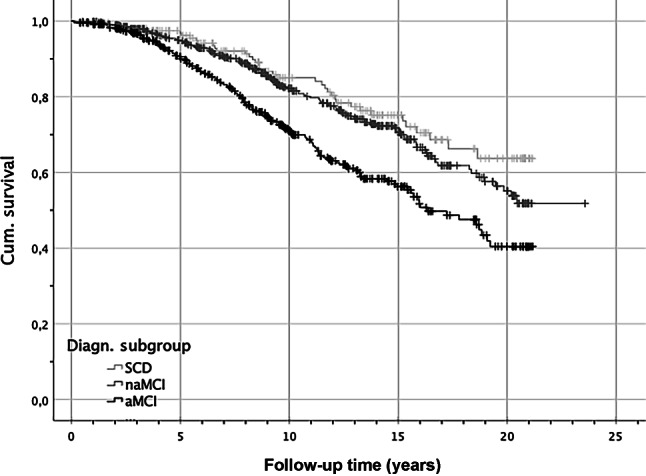
KM survival functions regarding the three diagnostic subgroups. *SCD* subjective cognitive decline, *naMCI* non-amnestic mild cognitive impairment, *aMCI* amnestic mild cognitive impairment

In the next step, the survival probability was assessed using the Kaplan-Meier method. Figure 2 illustrates the survival function after cognitive testing regarding the three diagnostic subgroups taking excluded cases into account. The five-year survival probability of SCD was 96.8%, naMCI was 94.7%, and aMCI was 90.6%. Log-rank testing revealed a significant difference between the three diagnostic subgroups, *p* < 0.001, considering all participants. Taking five-year survival functions into account, considering the Bonferroni adjustment (α* = 0.0167), pairwise comparisons indicated a significant difference only between SCD vs. aMCI, *p* = 0.009.

To evaluate the explanatory value of covariates and predictors regarding the mortality of patients within the observation period, a Cox-proportional regression was performed, as shown in Table 3. The influences of the predictors have to be interpreted as follows: depressiveness did not reveal a substantial contribution as a risk factor for mortality in all five model blocks, BDI-II with HR 0.997 [0.978; 1.02] and GDS-15 with HR 1.03 [0.98; 1.08], *p* ≥ 0.273. Conversely, increasing age with HR 1.09 [1.07; 1.11] as well as male gender with HR (inverted) 1.53 [1.17; 2.00] can be considered as risk factors in all five model blocks for higher mortality, *p*’s < 0.001. Similarly, an explanatory value was found for neurocognitive diagnoses up to the 4th model block; in particular, a significant risk for mortality was shown for aMCI vs. SCD diagnosis with HR 1.91 [1.33; 2.76], *p* < 0.001. After adding the six domains of the NTBV in the 5th and last model block, the two domains attention and perceptual speed with HR 1.34 [1.18; 1.53], *p* < 0.001, and executive functions with HR 1.24 [1.11; 1.39], *p* < 0.001, each had significant weight in explaining mortality. Accordingly, no negative influence on survival probability can be attributed to depressiveness. The influence of certain cognitive structures, specifically attention and perceptual speed, and executive functions is to be considered higher.

**Table 3:** Cox-regression modeling, coefficients of predictors considering the mortality criterion, five blocks (*n* = 1221)

Predictor	*B*	*SE*	Wald χ^2^ (df)	*p*-value	HR	95% CI HR	
						LB	UB
*Block 1*							
BDI-II	−0.004	0.010	0.184 (1)	0.668	0.996	0.977	1.015
GDS	0.016	0.024	0.417 (1)	0.519	1.016	0.969	1.065
*Block 1–2*							
BDI-II	−0.001	0.010	0.021 (1)	0.884	0.999	0.979	1.018
GDS	0.038	0.023	2.578 (1)	0.108	1.038	0.992	1.087
Age	0.106	0.008	198.537 (1)	<0.001^**^	1.112	1.096	1.129
Sex	−0.588	0.118	24.903 (1)	<0.001^**^	0.556	0.441	0.700
*Block 1–3*							
BDI-II	−0.003	0.010	0.075 (1)	0.784	0.997	0.978	1.017
GDS	0.040	0.024	2.918 (1)	0.088	1.041	0.994	1.090
Age	0.106	0.008	199.907 (1)	<0.001^**^	1.112	1.096	1.129
Sex	−0.636	0.122	27.192 (1)	<0.001^**^	0.529	0.417	0.672
School years	−0.003	0.018	0.029 (1)	0.865	0.997	0.962	1.033
WST-IQ	−0.006	0.005	1.643 (1)	0.200	0.994	0.985	1.003
*Block 1–4*							
BDI-II	−0.004	0.010	0.137 (1)	0.711	0.996	0.977	1.016
GDS	0.040	0.023	2.932 (1)	0.087	1.041	0.994	1.090
Age	0.106	0.008	193.906 (1)	<0.001^**^	1.111	1.095	1.128
Sex	−0.586	0.125	22.103 (1)	<0.001^**^	0.557	0.436	0.711
School years	−0.007	0.018	0.164 (1)	0.685	0.993	0.957	1.029
WST-IQ	−0.002	0.005	0.186 (1)	0.666	0.998	0.988	1.008
Diag. subgroup	–	–	15.641 (2)	<0.001^**^	–	–	–
naMCI vs. SCD	0.286	0.189	2.295 (1)	0.130	1.331	0.919	1.928
aMCI vs. SCD	0.649	0.186	12.150 (1)	<0.001^**^	1.913	1.328	2.755
*Block 1–5*							
BDI-II	−0.003	0.010	0.124 (1)	0.725	0.997	0.978	1.016
GDS	0.026	0.024	1.203 (1)	0.273	1.026	0.980	1.075
Age	0.087	0.009	86.827 (1)	<0.001^**^	1.091	1.071	1.111
Sex	−0.426	0.137	9.682 (1)	0.002	0.653	0.499	0.854
School years	0.003	0.019	0.020 (1)	0.887	1.003	0.966	1.040
WST-IQ	0.006	0.005	1.156 (1)	0.282	1.006	0.995	1.016
Diag. subgroup	–	–	1.532 (2)	0.465	–	–	–
naMCI vs. SCD	0.108	0.200	0.289 (1)	0.591	1.114	0.752	1.650
aMCI vs. SCD	0.278	0.240	1.344 (1)	0.246	1.321	0.825	2.114
*NTBV*							
1 attent. & perceptual speed	0.295	0.066	19,791 (1)	< 0.001^**^	1.343	1.179	1.529
2 verbal memory	−0.153	0.090	2.875 (1)	0.090	0.859	0.720	1.024
3 executive functions	0.217	0.056	14.917 (1)	< 0.001^**^	1.243	1.113	1.387
4 planning capacity	0.007	0.061	0.013 (1)	0.908	1.007	0.893	1.135
5 verbal fluency	−0.121	0.064	3.598 (1)	0.058	0.886	0.782	1.004
6 divergent reasoning	−0.038	0.058	0.425 (1)	0.514	0.963	0.859	1.079

*aMCI* amnestic mild cognitive impairment; *naMCI* non-amnestic mild cognitive impairment; *SCD* subjective cognitive decline; *BDI-II* Beck-Depressions-Inventar II; *GDS* Geriatric Depression Scale; *MMSE* Mini Mental State Examination; *WST-IQ* Wortschatztest-IQ; *NTBV* Neuropsychological Test Battery Vienna; *CI* confidence interval; *HR* hazard ratio

^**^p ≤ 0.01

## Discussion

Of research interest was the extent to which depressive symptoms influence the likelihood of survival in cognitively impaired patients such as SCD, naMCI, aMCI, as depression often appears in relation to cognitive decline in the senior population. In cognitively impaired patients, in turn, the risk of depressive symptoms is significantly increased. Due to the similar neuropathological changes and clinical manifestations, both syndromes often occur simultaneously in age [37]. The current point of view, according to the available literature, shows a greater mortality rate of older individuals with cognitive impairment [8–10]. The main result of the Cox Proportional Hazards model was that depressiveness did not reach a significant explanatory value and no negative influence on survival probability can be attributed. Laudisio et al. similarly did not find a generally increased mortality in older patients with depressive symptoms. However, depressive symptoms are a potentially reversible factor in increased hospitalization rates for these patients, regardless of the presence and seriousness of any other medical condition [38]. This suggests to us that depressiveness may have clinical relevance in the senior population. Furthermore, a recent study with comparable patient characteristics, obtained by Đapić et al., revealed that depressivity (GDS-15) was a significant predictor of five-year mortality in the patient population, suggesting that depressive symptoms may be considered a risk factor for mortality in patients with cognitive decline, *p* = 0.009, although the clinical effect is rated as low [39]. Data from this study also permit this interpretation. While depressiveness (GDS-15) showed at least a tendency to be considered as a risk factor for mortality up to the 4th model block, HR 1.04 [0.994; 1.09], *p* = 0.087, BDI-II did not reveal a substantial contribution. It should be mentioned that the GDS-15 is a screening examination of depressive mood and depression in the older population, whereas the BDI-II serves as an instrument for classifying the severity of depressive symptoms. The correlation between BDI-II and GDS-15 scoring appears to have a moderate to high relation, Pearson’s *r* (1221) = 0.72 (*p* < 0.001, two-tailed; 95%-CI [0.70; 0.75]). The curvilinear quadratic regression function *R*^2^ between BDI-II and GDS-15 attained 53.1%. This methodological approach leads to a multicollinearity of both factors.

The potential conversion of cognitive impairment to a more severe form should be mentioned. Mortality in some cases is not the final stage of progression in a cohort study. This means that the risk profile of these patients changes over time and is higher than that of patients without conversion. This is not reflected in the Cox proportional hazards model. Fine-Gray Competing Risks Regression is needed for a more detailed investigation. In addition, depressive symptomatology is a time-varying construct that changes in particular as patients move from one cognitive stage to a more advanced one. This means that changes in depressiveness may have a different impact on mortality in the future than at baseline. Characteristics of the depression parameters in each of the diagnostic subgroups did not reveal a significant difference in BDI-II data (*p* = 0.056) and in the GDS-15 (*p* = 0.062) in this report. Therefore, the extent of depressiveness may not be related to the severity of cognitive impairment. The varying relation between cognitive impairment and depression over time has already been addressed in the literature [14, 15].

Further studies are needed for closer estimation of the impact of depressive symptoms on mortality in patients with cognitive impairment. Depressive symptoms are thought to play the role of a mediator in explaining survival function. This view could be assessed by appropriate regression analytic mediator models. One possible clinical guideline is that older patients with cognitive impairment should be routinely screened for depressive symptoms. Such a routine screening would help identify patients with depressive symptoms more accurately in clinical practice and improve the quality of subsequent therapeutic interventions. It has been shown that depressive symptoms influence health-related quality of life in these patient groups. Thus, using health related quality of life as mediator variable in future studies would be worthwhile to pursue [40].

### Limitations

It should be noted that a number of individuals with cognitive impairment were not able to fully complete the tests, especially those for the assessment of depressiveness. These individuals were removed from further analysis due to the lack of data. Although it is conceivable that the exclusion of these patients might influence the results of the study we do not expect such an effect due to the small number of exclusions. Only complete data protocols were analyzed in the present study. This implies that protocols with missing values were not used, and no data imputations were performed.

## Conclusion

The results of the study suggest that depressiveness cannot be attributed a negative influence on the probability of survival, but the impact of certain cognitive structures, especially attention & perceptual speed, and executive functions, may be considered comparatively higher. At the same time, age as well as male gender act as a negative influence on survival in patients with cognitive impairment, whereas the diagnostic subgroup shows an impact only up to the last block using Cox modeling. This leads to the conclusion that the diagnostic subgroup categorization reveals a subordinate predictive role. NTBV subdomains appear to be valuable predictors of survival in patients with cognitive impairment, and further research on the predictive standing of neurocognitive ability is recommended. The same applies to depressiveness. Further studies are needed to understand the relationship between depressive symptomatology and cognitive decline, as well as to infer the influences on survival.

## Data Availability

The data that support the findings of this study are available from the corresponding author upon reasonable request.

## References

[1] Gómez-Gómez ME, Zapico SC. Frailty, cognitive decline, neurodegenerative diseases and nutrition interventions. Int J Mol Sci. 2019;20(11):2842. 31212645 10.3390/ijms20112842 PMC6600148

[2] Winblad B, Amouyel P, Andrieu S, Ballard C, Brayne C, Brodaty H, et al. Defeating Alzheimer’s disease and other dementias: a priority for European science and society. Lancet Neurol. 2016;15(5):455–532. 26987701 10.1016/S1474-4422(16)00062-4

[3] Petersen RC. Mild cognitive impairment as a diagnostic entity. J Intern Med. 2004;256(3):183–94. 15324362 10.1111/j.1365-2796.2004.01388.x

[4] Albert MS, DeKosky ST, Dickson D, Dubois B, Feldman HH, Fox NC, et al. The diagnosis of mild cognitive impairment due to Alzheimer’s disease: recommendations from the national institute on Aging-Alzheimer’s Association workgroups on diagnostic guidelines for Alzheimer’s disease. Alzheimers Dement. 2011;7(3):270–9. 21514249 10.1016/j.jalz.2011.03.008 PMC3312027

[5] Burmester B, Leathem J, Merrick P. Subjective cognitive complaints and objective cognitive function in aging: a systematic review and meta-analysis of recent cross-sectional findings. Neuropsychol Rev. 2016;26(4):376–93. 27714573 10.1007/s11065-016-9332-2

[6] Reid LM, MacLullich AMJ. Subjective memory complaints and cognitive impairment in older people. Dement Geriatr Cogn Disord. 2006;22(5–6):471–85. 17047326 10.1159/000096295

[7] Mitchell AJ, Beaumont H, Ferguson D, Yadegarfar M, Stubbs B. Risk of dementia and mild cognitive impairment in older people with subjective memory complaints: meta-analysis. Acta Psychiatr Scand. 2014;130(6):439–51. 25219393 10.1111/acps.12336

[8] James BD, Leurgans SE, Hebert LE, Scherr PA, Yaffe K, Bennett DA. Contribution of Alzheimer disease to mortality in the United States. Neurology. 2014;82(12):1045–50. 24598707 10.1212/WNL.0000000000000240 PMC3962992

[9] Perna L, Wahl HW, Mons U, Saum KU, Holleczek B, Brenner H. Cognitive impairment, all-cause and cause-specific mortality among non-demented older adults. Age Ageing. 2015;44(3):445–51. 25468013 10.1093/ageing/afu188

[10] Petersen JD, Waldorff FB, Siersma VD, Phung TKT, Bebe ACKM, Waldemar G. Major depressive symptoms increase 3‑year mortality rate in patients with mild dementia. Int J Alzheimers Dis. 2017;2017:1–8. 10.1155/2017/7482094 PMC5397625 28484660

[11] Gambassi G, Landi F, Lapane KL, Sgadari A, Mor V, Bernabei R. Predictors of mortality in patients with Alzheimer’s disease living in nursing homes. J Neurol Neurosurg Psychiatry. 1999;67(1):59–65. 10369823 10.1136/jnnp.67.1.59 PMC1736445

[12] Moser S. Cognitive functions and mortality in patients with subjective cognitive decline, mild cognitive impairment and Alzheimer’s disease. Wien: Medizinische Universität Wien; 2018.

[13] Steffens DC, Potter GG. Geriatric depression and cognitive impairment. Psychol Med. 2008;38(2):163–75. 17588275 10.1017/S003329170700102X

[14] Byers AL, Yaffe K. Depression and risk of developing dementia. Nat Rev Neurol. 2011;7(6):323–31. 21537355 10.1038/nrneurol.2011.60 PMC3327554

[15] Geerlings MI, den Heijer T, Koudstaal PJ, Hofman A, Breteler MMB. History of depression, depressive symptoms, and medial temporal lobe atrophy and the risk of Alzheimer disease. Neurology. 2008;70(15):1258–64. 18391157 10.1212/01.wnl.0000308937.30473.d1

[16] Jessen F, Amariglio RE, Boxtel M, Breteler M, Ceccaldi M, Chételat G, et al. A conceptual framework for research on subjective cognitive decline in preclinical Alzheimer’s disease. Alzheimers Dement. 2014;10(6):844–52. 24798886 10.1016/j.jalz.2014.01.001 PMC4317324

[17] Goldmann U, Roth E, Schaub A. Kognitiv-psychoedukative Therapie zur Bewältigung von Depressionen. 2nd ed. Hogrefe; 2013. p. 174.

[18] Beck AT, Hautzinger M, editors. Beck-Depressions-Inventar: BDI. 2nd ed. Bern, Göttingen: Huber; 2001.

[19] Deutsche Gesellschaft für Psychiatrie und Psychotherapie, Psychosomatik und Nervenheilkunde (DGPPN), Bundesärztekammer (BÄK), Kassenärztliche Bundesvereinigung (KBV), Arbeitsgemeinschaft der Wissenschaftlichen Medizinischen Fachgesellschaften (AWMF). S3-Leitlinie/Nationale VersorgungsLeitlinie Unipolare Depression – Langfassung. 2015. https://www.leitlinien.de/themen/depression/pdf/depression-2aufl-vers5-lang.pdf. Accessed 10 Feb 2022.

[20] Yesavage JA, Brink TL, Rose TL, Lum O, Huang V, Adey M, et al. Development and validation of a geriatric depression screening scale: a preliminary report. J Psychiatr Res. 1982;17(1):37–49. 7183759 10.1016/0022-3956(82)90033-4

[21] Creavin ST, Wisniewski S, Noel-Storr AH, Trevelyan CM, Hampton T, Rayment D, et al. Mini-mental state examination (MMSE) for the detection of dementia in clinically unevaluated people aged 65 and over in community and primary care populations. 2016. https://doi.wiley.com/10.1002/14651858.CD011145.pub2. Accessed 16 Feb 2022. 10.1002/14651858.CD011145.pub2 PMC8812342 26760674

[22] Nilsson FM. Mini mental state examination (MMSE)—probably one of the most cited papers in health science. Acta Psychiatr Scand. 2007;116(2):156–7. 17650282 10.1111/j.1600-0447.2007.01037.x

[23] Schmidt KH, Metzler P. Wortschatztest: WST. Weinheim: Beltz; 1992.

[24] Lehrner J, Maly J, Gleiss A, Auff E, Dal-Bianco P. The Vienna neurophysiological test battery (VNTB) for detecting Alzheimer’s dementia: standardization, norms, validation. 2007. https://www.researchgate.net/profile/Johann-Lehrner/publication/272825680_The_Neuropsychological_Test_Battery_Vienna_NTBV/links/54f070de0cf2432ba65b23ee/The-Neuropsychological-Test-Battery-Vienna-NTBV.pdf. Accessed 16 Feb 2022.

[25] Manual neuropsychological test battery Vienna. 2022. www.psimistri.com.

[26] Weiß C. Basiswissen Medizinische Statistik. Berlin, Heidelberg: Springer; 2019. 10.1007/978-3-662-56588-9.

[27] Field A. Discovering statistics using IBM SPSS statistics. 4th ed. London: SAGE; 2013.

[28] Kubinger KD, Rasch D, Moder K. Zur Legende der Voraussetzungen des t‑Tests für unabhängige Stichproben. Psychol Rundsch. 2009;60(1):26–7. 10.1026/0033-3042.60.1.26

[29] Ziegler A, Lange S, Bender R. Überlebenszeitanalyse: Der Log-Rang-Test. Dtsch Med Wochenschr. 2007;132(1):e39–41. 17530595 10.1055/s-2007-959040

[30] Bühl A. SPSS 20: Einführung in die moderne Datenanalyse. 13th ed. München: Pearson; 2012. p. 1049.

[31] Ziegler A, Lange S, Bender R. Überlebenszeitanalyse: Die Cox-Regression. Dtsch Med Wochenschr. 2007;132(1):e42–4. 17530596 10.1055/s-2007-959039

[32] Hatzinger R, Nagel H. PASW Statistics: statistische Methoden und Fallbeispiele. München: Pearson Studium; 2009. p. 352.

[33] Moosbrugger H, Kelava A. Testtheorie und Fragebogenkonstruktion. 2nd ed. Berlin: Springer; 2012.

[34] Bortz J, Schuster C. Statistik für Human- und Sozialwissenschaftler. Limitierte Sonderausgabe. 7th ed. Berlin, Heidelberg: Springer; 2016. p. 655.

[35] Kubinger KD. Psychologische Diagnostik: Theorie und Praxis psychologischen Diagnostizierens. 3rd ed. Göttingen: Hogrefe; 2019. p. 553.

[36] Wirtschaftskammer Österreichs. Altersstruktur der Bevölkerung 2022. 2022. https://wko.at/statistik/bundesland/Altersstruktur.pdf. Accessed 11 Nov 2022.

[37] World Health Organisation. International statistical classification of diseases and related health problems. 10th ed. World Health Organization; 2007.

[38] Laudisio A, Marzetti E, Pagano F, Pozzi G, Bernabei R, Zuccalà G. Depressive symptoms are associated with hospitalization, but not with mortality in the elderly: a population-based study. Aging Ment Health. 2010;14(8):955–61. 21069601 10.1080/13607863.2010.501058

[39] Đapić B, Schernhammer E, Haslacher H, Stögmann E, Lehrner J. No effect of thyroid hormones on 5‑year mortality in patients with subjective cognitive decline, mild cognitive disorder, and Alzheimer’s disease. J Neuroendocrinol. 2022; 10.1111/jne.13107. 35213057 10.1111/jne.13107 PMC9286816

[40] Pusswald G, Moser D, Pflüger M, Gleiss A, Auff E, Stögmann E, Dal-Bianco P, Lehrner J. The impact of depressive symptoms on health-related quality of life in patients with subjective cognitive decline, mild cognitive impairment, and Alzheimer’s disease. Int Psychogeriatr. 2016;28(12):2045–54. 10.1017/S1041610216001289. 27576786 10.1017/S1041610216001289

